# Metabolic stress and macrophage efferocytosis in osteoarthritis: mechanisms and therapeutic implications

**DOI:** 10.3389/fimmu.2026.1999085

**Published:** 2026-10-12

**Authors:** HaoYang Wu, DeChao Yuan, RenJian He, ShiWei Liu

**Affiliations:** Department of Orthopedics, Zigong First People’s Hospital, Zigong, China

**Keywords:** efferocytosis, immunometabolism, inflammation resolution, lactylation, metabolic stress, osteoarthritis, synovial macrophages

## Abstract

Metabolic disturbances contribute to osteoarthritis through mechanisms that extend beyond increased mechanical loading, yet their effects on inflammatory resolution remain incompletely understood. Macrophage efferocytosis—the recognition, engulfment, and degradation of apoptotic cells—provides a potential link between metabolic stress and persistent synovitis. This Mini Review examines how obesity and hyperglycemia disrupt synovial macrophage clearance and evaluates the rationale for therapeutic restoration. Human observations and experimental osteoarthritis studies implicate reduced growth arrest-specific 6 signaling and hyperglycemia-associated CD11b lactylation in defective apoptotic-cell recognition and uptake. Complementary evidence from other inflammatory conditions identifies membrane trafficking, mitochondrial adaptation, and lysosomal cargo processing as additional determinants of clearance competence. Importantly, glycolysis and lactate have context-dependent effects: although lactylation can impair recognition, adaptive lactate signaling supports repeated engulfment and expansion of pro-resolving macrophages. This distinction challenges indiscriminate glycolytic inhibition and favors interventions matched to the affected clearance step. Defective efferocytosis may amplify synovitis through persistence of cellular debris and loss of resolution-promoting signals, thereby creating an environment unfavorable to cartilage homeostasis. Emerging receptor-directed, metabolic, and intra-articular delivery strategies offer opportunities to restore clearance, but clinical validation remains necessary. Translation will require identification of susceptible macrophage populations, functional assessment of the complete efferocytic process, and demonstration that clearance recovery mediates meaningful improvements in joint structure and symptoms.

## 1 Introduction

Osteoarthritis (OA) is a heterogeneous whole-joint disease in which mechanical injury, metabolic disturbances, and inflammation interact to produce progressive tissue dysfunction and disability (1). Obesity-associated OA involves changes in metabolic and inflammatory signaling alongside increased joint loading, although the contribution of individual metabolic abnormalities varies across patient populations and joint sites (2). The synovium provides an important interface for these interactions. Resident and recruited cells respond to tissue damage and metabolic cues by generating inflammatory signals associated with structural deterioration and pain (3). Understanding why these responses persist is therefore central to identifying actionable immune mechanisms in OA.

Synovial macrophages are particularly relevant because their functions encompass inflammatory signaling, tissue surveillance, and clearance of dying cells. In experimental OA, obesity increases the early influx of inflammatory monocyte-derived macrophages after injury and compromises the subsequent recovery of barrier-forming synovial lining macrophages (4). These findings raise the possibility that metabolic stress sustains synovitis partly by disrupting macrophage homeostatic functions. Efferocytosis—the recognition, engulfment, and degradation of apoptotic cells—is one such function. Experimental work outside the joint demonstrates that processing apoptotic-cell cargo can activate metabolic and transcriptional programs that promote inflammation resolution, providing a mechanistic basis for investigating this process in OA (5).

Direct evidence of defective efferocytosis has emerged from human OA. Synovial macrophages isolated from patients with late-stage knee OA exhibit reduced apoptotic-cell clearance, while OA synovial fluid can impair efferocytosis by blood-derived macrophages ex vivo (6). Studies combining patient samples with experimental models further implicate reduced growth arrest-specific 6 (GAS6) signaling in obesity-associated efferocytosis defects (7). More recently, hyperglycemia-associated glycolytic changes and CD11b lactylation have been linked to impaired recognition and clearance of apoptotic cells (8). Together, these observations suggest that distinct metabolic disturbances may converge on defective clearance. However, they do not establish whether efferocytosis failure initiates human OA or primarily amplifies an existing inflammatory environment.

This Mini Review examines how obesity and hyperglycemia alter synovial macrophage efferocytosis and evaluates the evidence connecting these defects to persistent inflammation and joint damage. We distinguish human observations from experimental causality, compare the functional steps affected by different metabolic disturbances, and assess the therapeutic rationale for restoring clearance. Particular attention is given to disease stage, reversibility, and the evidence required to translate improved efferocytosis into meaningful joint protection.

## 2 Macrophage efferocytosis in the osteoarthritic synovium

Efferocytosis links apoptotic-cell removal to the maintenance of tissue homeostasis. Its completion requires coordinated recognition, internalization, and lysosomal degradation, followed by adaptation to the ingested cargo. Exposed phosphatidylserine enables apoptotic-cell recognition through receptors that bind directly or engage bridging molecules, including GAS6 and protein S. Receptor signaling subsequently coordinates cytoskeletal remodeling and engulfment (9). These stages are functionally separable. A genome-wide CRISPR screen identified WDFY3 as a regulator of apoptotic-cell uptake and degradation. WDFY3 deficiency impaired engulfment without reducing initial binding (10). Consequently, receptor expression or target-cell attachment alone cannot establish effective clearance.

Successful efferocytosis also modifies macrophage behavior after engulfment. Sukka et al. demonstrated that apoptotic-cell processing activates an IDO1-dependent tryptophan–kynurenine–aryl hydrocarbon receptor pathway, supporting interleukin-10 production and further efferocytosis (11). This coupling of cargo processing to a pro-resolving response provides a rationale for examining clearance failure in persistent synovitis. However, these mechanisms were established in macrophage systems and mouse models outside OA; their activity and significance in human synovial macrophages remain to be determined.

The strongest direct human evidence comes from functional studies of knee OA. Del Sordo et al. identified increased apoptotic-cell burden in late-stage OA synovium, together with fewer macrophages participating in efferocytosis and reduced uptake per macrophage. OA synovial fluid also suppressed clearance by blood-derived macrophages, with the greatest inhibition elicited by samples from patients with early-stage disease and higher pain and inflammation (6). These findings implicate the local joint environment and suggest that clearance dysfunction does not simply track radiographic severity. Nevertheless, assays using apoptotic Jurkat cells do not identify which endogenous synovial cell populations escape removal, and the available cross-sectional observations cannot establish temporal causality.

Macrophage senescence adds another potential determinant of clearance capacity. Experimental OA studies associate senescent synovial macrophages with mitochondrial injury, inflammatory secretion, and impaired efferocytosis; interventions targeting these cells improve clearance-related measures and joint pathology in mice (12). Because these interventions affect several cellular processes, their benefits cannot be assigned exclusively to efferocytosis. Functional assessment should therefore distinguish recognition, uptake, and degradation while accounting for macrophage state and apoptotic-cell availability. Neither M1/M2 marker expression nor apoptotic-cell accumulation alone resolves these distinctions. Establishing the affected clearance step is essential for determining how obesity and hyperglycemia disrupt synovial homeostasis.

## 3 Metabolic stress as a driver of defective macrophage efferocytosis

Metabolic stress can disrupt efferocytosis at several interdependent stages: recognition of apoptotic cells, membrane remodeling during engulfment, intracellular cargo processing, and adaptation to repeated uptake. In OA, GAS6 deficiency and CD11b lactylation provide direct mechanistic links between metabolic disturbances and impaired clearance. Complementary evidence from other inflammatory settings identifies additional processes that may determine whether synovial macrophages accommodate increased clearance demands or lose their capacity to resolve inflammation (**Figure 1**).

**Figure 1:**
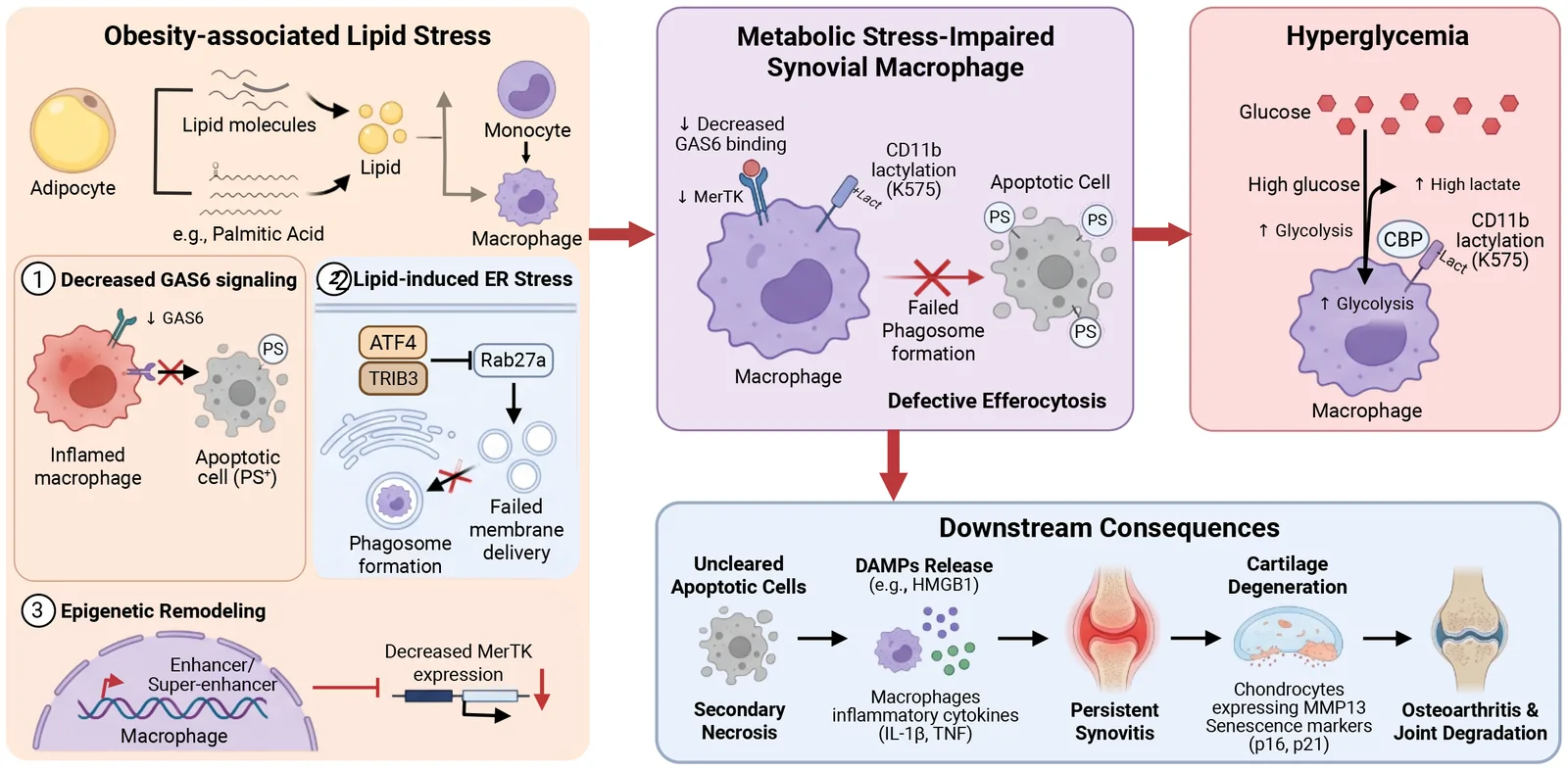
Summarizes selected mechanisms through which metabolic stress may impair macrophage efferocytosis and contribute to persistent synovitis and cartilage degeneration. Schematic overview of selected mechanisms linking metabolic stress to impaired macrophage clearance of apoptotic cells and joint inflammation. Obesity-associated lipid stress and hyperglycemia are considered upstream metabolic disturbances that may compromise efferocytosis through distinct pathways. In obesity-associated osteoarthritis (OA), reduced macrophage secretion of growth arrest-specific 6 (GAS6) limits apoptotic-cell recognition and clearance. Complementary studies in non-joint experimental systems indicate that lipid-induced endoplasmic reticulum stress activates the ATF4–TRIB3 pathway, impairing Rab27a-dependent membrane delivery and phagosome closure. Palmitic acid can also remodel enhancers and super-enhancers associated with efferocytosis-related genes, including reduced MERTK expression. In hyperglycemia-associated OA, increased macrophage glycolysis and lactate accumulation promote CBP-mediated CD11b lactylation at lysine 575 (K575), impairing apoptotic-cell recognition and uptake. These disturbances may converge on defective efferocytosis, allowing uncleared apoptotic cells to undergo secondary necrosis and release damage-associated molecular patterns (DAMPs). The resulting inflammatory environment may sustain macrophage cytokine production, persistent synovitis, and cartilage degeneration. Increased MMP13 expression and senescence-associated markers illustrate potential downstream chondrocyte responses. The diagram integrates OA-related findings with mechanisms established outside the joint; the ATF4–TRIB3–Rab27a and enhancer-remodeling pathways require validation in OA synovial macrophages. The downstream sequence represents a proposed pathogenic framework rather than a uniformly established causal cascade.

### 3.1 Obesity-associated lipid stress: from GAS6 deficiency to defective recognition and engulfment

Obesity-associated OA illustrates how increased macrophage abundance can coexist with insufficient clearance capacity. Yao et al. identified reduced GAS6 expression together with inflammatory macrophage accumulation and increased apoptotic-cell burden in obese OA synovium. Mechanistic experiments linked inflammatory macrophage activation to diminished GAS6 secretion and impaired efferocytosis. Recombinant GAS6 enhanced apoptotic-cell uptake, whereas AXL inhibition attenuated this response. Intra-articular GAS6 administration also reduced apoptotic-cell accumulation, synovitis, and cartilage damage in experimental OA, supporting deficient ligand availability as a potentially reversible component of failed clearance. However, the animal comparison combined Apoe deficiency, a high-fat diet, and surgical joint destabilization, limiting attribution of the phenotype to adiposity alone. The specific metabolic signals responsible for GAS6 suppression therefore remain unresolved (7).

Recent work provides evidence that lipid exposure can alter the transcriptional programs supporting efferocytosis. In human monocytes, palmitic acid remodeled enhancers and super-enhancers associated with inflammatory and efferocytosis-related genes. These changes were accompanied by increased inflammatory gene expression, reduced expression of clearance-associated genes, and impaired efferocytosis in human macrophages (13). This study supports an epigenetic route from lipid excess to clearance dysfunction, although it does not establish that the same regulatory changes account for GAS6 deficiency in OA.

Lipid stress can also impair engulfment after apoptotic cells have been encountered. Singhal et al. demonstrated that lipid-induced endoplasmic reticulum stress activates an ATF4–TRIB3 pathway that suppresses Rab27a-dependent focal exocytosis. Reduced delivery of intracellular membrane to developing phagosomes delays phagosome closure and compromises efferocytosis. Genetic and pharmacological interventions restored clearance in experimental settings, while macrophages from carriers of a gain-of-function TRIB3 variant exhibited reduced efferocytic capacity (14). These findings identify membrane supply as a distinct metabolic vulnerability: enhancing apoptotic-cell recognition may be insufficient when the machinery required to complete engulfment remains defective.

Recognition pathways themselves are also susceptible to prolonged metabolic inflammation. In experimental steatohepatitis, hypernutrition-induced TNF and IL-1β promoted ADAM17-dependent TREM2 shedding, disrupting clearance of lipid-laden apoptotic hepatocytes (15). A separate study implicated loss of macrophage TIM4 in defective hepatocyte clearance and fibrogenesis; restoring TIM4 enhanced efferocytosis and reduced fibrosis through a mechanism involving macrophage-derived IL-10 (16). These liver studies do not establish TREM2 or TIM4 dysfunction in OA. They nevertheless support investigating whether obesity-associated synovial clearance failure combines reduced bridging-molecule availability with altered receptor expression, receptor shedding, and defective membrane trafficking.

### 3.2 Hyperglycemia and glycolytic remodeling: pathogenic lactylation versus adaptive lactate signaling

Hyperglycemia can compromise efferocytosis through modification of the recognition machinery. Zhou et al. linked increased macrophage glycolysis and lactate accumulation to CD11b lactylation at lysine 575 in hyperglycemia-associated OA. Functional experiments demonstrated impaired apoptotic-cell recognition and uptake. Molecular simulations suggested that lactylation alters CD11b conformation and its interaction with phosphatidylserine. Targeted knockdown of CREB-binding protein (CBP), identified as a mediator of CD11b lactylation, reduced the modification, restored efferocytosis, and attenuated experimental OA. These findings connect glucose metabolism to receptor function through a non-histone post-translational modification. Nevertheless, the human observations were limited to end-stage OA. Causal evidence came from high-glucose cultures and a mouse model combining streptozotocin-induced diabetes with surgical OA. Validation in earlier disease and in models incorporating insulin resistance remains necessary (8).

Evidence from atherosclerosis further indicates that particular glycolytic regulators can couple inflammatory activation to clearance dysfunction. Myeloid PKM2 deletion reduced glycolytic activity and enhanced LRP1-associated efferocytosis; silencing LRP1 diminished this improvement. Limiting PKM2 nuclear translocation also enhanced clearance and reduced experimental atherosclerosis (17). These observations support targeting defined metabolic regulatory mechanisms, but they do not establish that suppression of glycolysis will universally improve macrophage function.

Indeed, apoptotic-cell uptake can induce a glycolytic program required for continued clearance. Schilperoort et al. showed that initial engulfment activates PFKFB2-dependent glycolysis, generating intracellular lactate that promotes calcium-dependent increases in cell-surface MerTK and LRP1. This adaptation supports uptake of subsequent apoptotic cells, and disruption of PFKFB2 activation impaired clearance in a thymocyte-apoptosis model (18). Thus, an increase in glycolytic flux can sustain efferocytosis when coordinated with the demands imposed by prior engulfment.

Lipid mediators provide another connection between cargo processing and this adaptive response. Breakdown of apoptotic cells increased macrophage production of maresin conjugates in tissue regeneration (MCTRs), which promoted continual efferocytosis through Rac1-dependent changes in glucose uptake and glycolytic metabolism. Inhibiting glycolysis curtailed the clearance and repair responses supported by these mediators (19). Lactate also supports expansion of the resolving macrophage population. Efferocytosis-induced lactate signaling through GPR132 stabilizes Myc. This pathway cooperates with signals from apoptotic-cell-derived nucleotides to enable macrophage proliferation (20).

These results expose a central unresolved issue in metabolic OA: under which conditions does glucose-derived lactate support clearance, and when does it favor inhibitory receptor modification? Exposure duration, metabolic state, receptor availability, and the stage of efferocytosis examined are plausible determinants, but their relative contributions have not been resolved in matched experiments using OA synovial macrophages. The discrepancy also cannot be explained simply by assigning harmful effects to intracellular lactate, because intracellular lactate supports continual efferocytosis in the PFKFB2 pathway. Consequently, lactate abundance or glycolytic activity alone is unlikely to identify clearance-defective macrophages. Functional measurements must distinguish initial uptake, repeated engulfment, and proliferation of efferocytic populations.

### 3.3 Mitochondrial and lysosomal constraints on sustained efferocytosis

Successful recognition and engulfment must be supported by organelle functions that accommodate the energetic and degradative demands of apoptotic-cell uptake. Evidence directly relevant to the joint comes from experimental temporomandibular joint OA. Dental pulp stem cell treatment enhanced macrophage efferocytosis and reduced synovitis, accompanied by increased PINK1/Parkin-associated mitophagy. Pharmacological inhibition of mitochondrial quality-control processes partially attenuated the macrophage response (21). This study supports an association between mitochondrial maintenance and synovial clearance competence. However, its chemically induced rat model and cell-based intervention do not establish a specific PINK1/Parkin defect caused by obesity or hyperglycemia in knee OA.

Mechanistic studies outside the joint show why mitochondrial support extends beyond ATP production alone. Merlin et al. demonstrated that GLS1-dependent glutamine metabolism sustains efferocytosis through a non-canonical transaminase pathway coupling redox buffering to oxidative phosphorylation. This coordination meets the requirements for cellular detoxification and energy-intensive cytoskeletal remodeling; disrupting it impaired clearance and aggravated experimental atherosclerosis (22). These findings suggest that assessing antioxidant capacity together with respiratory function may be more informative than measuring either process in isolation.

Mitochondrial membrane potential also requires context-dependent interpretation. Dunphy et al. identified an IFNAR–ISG15 pathway that decreased membrane potential while preserving respiration and increasing ATP production, thereby enhancing macrophage efferocytosis (23). This adaptive response demonstrates that reduced membrane potential need not indicate mitochondrial failure. Its relevance to OA remains untested, but it cautions against inferring clearance dysfunction from a single mitochondrial readout without measuring engulfment and metabolic performance.

Lysosomal processing creates a further distinction between apparent uptake and completed clearance. In experimental liver injury, myeloid deficiency of the lysosomal cholesterol transporter NPC1 impaired apoptotic cargo degradation despite largely preserved binding and initial uptake. This defect accompanied increased inflammation and fibrosis (24). Although NPC1 dysfunction has not been established in OA synovial macrophages, the study illustrates how intracellular retention of engulfed material can conceal a clearance defect when assays measure only cargo-positive cells.

These observations support a working model in which metabolic stress produces several potentially coexisting limitations: deficient apoptotic-cell recognition, inadequate membrane delivery, insufficient metabolic adaptation, and delayed cargo degradation. For OA, the next mechanistic step is to determine whether these defects occur in the same macrophage populations and whether correcting one restores the entire clearance sequence. Such distinctions will be essential for identifying interventions that recover sustained efferocytosis while preserving the metabolic programs required for inflammation resolution.

## 4 From defective efferocytosis to persistent synovitis and cartilage degeneration

Defective efferocytosis may sustain joint inflammation through two complementary processes: persistence of inflammatory cellular material and failure to activate macrophage programs that normally promote resolution. This distinction extends the pathogenic significance of impaired clearance beyond apoptotic-cell accumulation. In metabolic OA, the resulting imbalance could connect the macrophage defects described above to continued synovitis and deterioration of the cartilage microenvironment.

When apoptotic cells remain uncleared, secondary necrosis can expose intracellular danger signals that reinforce inflammation. Such signals may themselves inhibit subsequent clearance, creating a feedback mechanism that perpetuates the original defect. In experimental acute lung injury, extracellular high-mobility group box 1 (HMGB1) suppressed macrophage Rab43 expression and impaired cell-surface delivery of the efferocytic receptor CD91. Myeloid Rab43 deficiency consequently delayed inflammatory resolution and aggravated tissue injury (25). These findings provide a mechanistic example of how extracellular damage signals can disable the machinery required for their removal. Whether this HMGB1–Rab43–CD91 pathway operates in OA synovium remains unresolved, but it offers a testable explanation for persistent clearance failure after the initiating metabolic insult.

Impaired efferocytosis may also deprive macrophages of signals that actively terminate inflammation. Efferocytosis-induced IDO1–kynurenine–AhR signaling promotes IL-10 expression and further apoptotic-cell clearance in experimental models of tissue resolution (11). Failure to engage this response could therefore reduce both anti-inflammatory output and the capacity to accommodate additional dying cells. Evidence relevant to OA comes from a proefferocytic microsphere study in which enhanced macrophage clearance was accompanied by Pbx1/IL-10 signaling, an anti-inflammatory macrophage phenotype, and improved joint outcomes (26). Because the intervention combined macrophage recruitment, targeted apoptosis, and efferocytosis enhancement, these results support coordinated restoration of inflammatory homeostasis without establishing the independent contribution of each component.

Macrophage–chondrocyte communication provides an experimental connection between clearance dysfunction and cartilage damage. In the GAS6 study, conditioned medium from macrophages exposed to apoptotic cells increased chondrocyte MMP13 and senescence-associated p16 and p21 expression while reducing type II collagen; GAS6 supplementation partially attenuated these changes (7). Thus, altered macrophage handling of apoptotic material can influence chondrocyte phenotype through soluble mediators, without requiring direct macrophage access to chondrocytes embedded within intact cartilage.

Cellular senescence may reinforce this interaction, although senescent cells must be distinguished from apoptotic efferocytic targets. Xiong et al. induced apoptosis in senescent chondrocytes using ABT263-containing liposomes and demonstrated subsequent macrophage uptake *in vitro*. Their lesion-targeted delivery system reduced senescence-associated cartilage pathology and improved repair in experimental OA (27). These findings support coupling senescent-cell elimination with effective clearance, rather than assuming that viable senescent chondrocytes are routinely removed by efferocytosis. Conversely, senescent fibroblasts and epithelial cells can suppress macrophage clearance of neighboring apoptotic cells. This inhibition involves contact-dependent CD47–SIRPα signaling associated with increased QPCT/L activity and occurs independently of the senescence-associated secretory phenotype (28). Although this mechanism awaits validation in joint tissues, it suggests a reciprocal relationship in which senescence both results from an unfavorable inflammatory environment and further compromises local clearance.

Finally, increased engulfment does not necessarily establish successful inflammatory resolution. In models of TNF-induced systemic inflammation and septic peritonitis, TNF-activated phagocytes underwent caspase-8-dependent pyroptosis and IL-1β maturation after engulfing neutrophils. These responses occurred independently of the NLRP3 inflammasome (29). This response has not been demonstrated in OA, but it identifies an important distinction between apoptotic-cell uptake and its immunological outcome. Accordingly, restoration of synovial efferocytosis should be evaluated through completed clearance, macrophage survival, inflammatory mediator production, and cartilage protection. These outcomes will help determine whether an intervention interrupts the proposed cycle of cellular damage, failed resolution, and progressive joint degeneration.

## 5 Therapeutic restoration of efferocytosis: targeted interventions and clinical translation

Restoring macrophage efferocytosis offers a potential means of interrupting the interaction between metabolic stress and persistent joint inflammation. Therapeutic development should address the dominant clearance defect, ensure delivery to the relevant joint compartment, and establish that improved engulfment translates into completed clearance and tissue protection. The OA-directed interventions discussed below remain preclinical, while emerging clinical studies provide useful benchmarks for metabolic treatment and intra-articular delivery (**Table 1**).

**Table 1:** Key evidence linking metabolic stress and macrophage efferocytosis to osteoarthritis.

Study and model	Pathway or clearance step	Main finding	Relevance and limitation
Del Sordo et al. (2023) (6) Human knee OA synovium; synovial-fluid exposure ex vivo.	Apoptotic-cell uptake	OA macrophages showed reduced uptake of apoptotic Jurkat cells. OA synovial fluid reproduced the defect in blood-derived macrophages.	Direct human functional evidence. Cross-sectional design; endogenous target cells and metabolic drivers remain unresolved.
Yao et al. (2023) (7) Human OA synovium; Apoe-deficient mice receiving a high-fat diet and surgical OA.	GAS6–AXL; recognition and uptake	Reduced GAS6 secretion accompanied impaired efferocytosis. GAS6 supplementation improved clearance and experimental joint pathology.	Direct metabolic OA evidence. Combined genetic, dietary and surgical exposures limit attribution to obesity alone.
Zhou et al. (2026) (8) Human OA samples; high-glucose cultures; streptozotocin plus surgical OA in mice.	CD11b K575 lactylation; recognition and uptake	Hyperglycemia increased CD11b lactylation and impaired clearance. Targeted CBP knockdown reduced lactylation and improved efferocytosis and experimental OA.	Direct metabolic OA evidence. End-stage human samples and a diabetes model that does not reproduce typical insulin resistance.
Liu et al. (2026) (12) Trauma- and aging-induced mouse OA; macrophage assays.	Senescence; STAT3–ADAM17–MerTK signaling	Chloroquine-bearing nanoparticles delivering SOD reduced macrophage stress, improved efferocytosis and alleviated experimental OA.	Direct OA intervention evidence. Multiple actions prevent exclusive attribution of protection to efferocytosis restoration.
Ma et al. (2025) (21) Chemically induced rat temporomandibular OA; macrophage cocultures.	PINK1/Parkin-associated mitophagy; uptake competence	Dental pulp stem cells enhanced mitophagy-associated measures and apoptotic-cell uptake while reducing synovitis. Pharmacological inhibition partly attenuated the response.	Joint-specific experimental evidence. Does not establish pathway-specific causality or a metabolic defect in knee OA.
Wang et al. (2025) (26) Macrophage assays and experimental OA.	Local efferocytosis enhancement; Pbx1/IL-10 signaling	Microspheres combined macrophage recruitment, targeted apoptosis and enhanced efferocytosis, accompanied by anti-inflammatory responses and improved joint outcomes.	Direct OA intervention evidence. Altered cell recruitment and survival confound the independent contribution of clearance.
Xiong et al. (2024) (27) Chondrocyte–macrophage assays; cartilage explants; experimental OA.	Senolysis followed by apoptotic-cell clearance	ABT263 induced senescent-chondrocyte apoptosis and subsequent macrophage uptake *in vitro*. Lesion-localized microspheres improved cartilage outcomes *in vivo*.	Direct OA intervention evidence. Viable senescent cells are not apoptotic targets; co-delivered PDGF-BB also supports repair.
Singhal et al. (2025) (14) Lipid-loaded macrophages; obese and atherosclerotic mice; human macrophages.	ATF4–TRIB3–Rab27a; membrane supply and phagosome closure	Lipid-induced endoplasmic reticulum stress reduced focal exocytosis and delayed closure. Disrupting ATF4/TRIB3 signaling restored efferocytosis in experimental systems.	Mechanistic support outside OA. Synovial activation of this pathway and its contribution to joint disease remain untested.
Schilperoort et al. (2023) (18) Macrophage assays; mouse thymocyte-apoptosis model.	PFKFB2–lactate; continual efferocytosis	Initial uptake induced glycolysis and intracellular lactate, increasing surface MerTK/LRP1 and supporting subsequent engulfment. Impaired PFKFB2 activation reduced clearance.	Adaptive mechanism outside OA. Demonstrates why glycolytic inhibition may compromise clearance; relevance to OA requires testing.
Guan et al. (2025) (24) Mouse liver injury; myeloid Npc1 deletion; macrophage assays.	NPC1; lysosomal cargo processing	Npc1 deficiency impaired apoptotic-cargo degradation despite largely preserved binding and initial uptake, accompanying greater liver inflammation and fibrosis.	Mechanistic support outside OA. NPC1 dysfunction in synovial macrophages has not been established.

Evidence categories describe relevance to OA, not a formal certainty assessment. Improved uptake or joint pathology alone does not establish complete cargo degradation or prove that efferocytosis mediates therapeutic benefit. Reference numbers correspond to the manuscript bibliography; linked numbers open PubMed records.

### 5.1 Restoring recognition and macrophage functional competence

The most direct strategies target mechanisms already implicated in metabolic OA. Intra-articular GAS6 supplementation improved apoptotic-cell clearance and reduced joint pathology in an obesity-associated OA model, supporting replacement of a deficient bridging molecule (7). In hyperglycemia-associated OA, targeted CBP knockdown reduced CD11b lactylation, restored macrophage efferocytosis, and attenuated experimental disease (8). These approaches suggest that ligand insufficiency and metabolically altered receptor function may require different interventions. Their prioritization should therefore depend on functional evidence of the corresponding defect, rather than obesity or diabetes status alone.

Restoring recognition may also require simultaneous correction of macrophage dysfunction. Chloroquine-bearing polymer nanoparticles delivering superoxide dismutase combined suppression of mitochondrial damage and senescence-associated signaling with improved efferocytosis through the STAT3/ADAM17/MerTK pathway. Intra-articular treatment alleviated both trauma- and aging-induced OA in mice (12). This study supports coordinated targeting of macrophage stress and clearance capacity, although the relative contributions of the nanoparticle components require further separation.

Engineering efferocytic receptors offers another experimental approach. Chimeric receptors combining phosphatidylserine-recognition domains with ELMO1 signaling enhanced apoptotic-cell uptake and reduced tissue injury in several inflammatory models. However, protection depended on the recipient cell type: expression in tissue epithelial cells was beneficial, whereas macrophage-lineage expression did not reproduce protection in the tested models (30). Extension to OA would therefore require direct testing in synovial macrophages, including whether enhanced uptake remains coupled to cargo degradation and anti-inflammatory output.

### 5.2 Matching intra-articular delivery to the therapeutic target

Local administration can improve joint exposure, but therapeutic targeting requires distinguishing synovial macrophages from cartilage-resident cells. A CXCR6-enriched membrane-coated nanocarrier increased delivery of capsaicin to CXCL16-expressing macrophages, prolonged synovial retention, and reduced experimental synovitis and cartilage degeneration (31). Although this study did not establish efferocytosis restoration, it provides a delivery strategy that could be evaluated with cargo designed to correct macrophage clearance defects.

Cartilage-directed platforms address a different anatomical objective. Disease-responsive anionic nanoparticles exploited glycosaminoglycan depletion to preferentially accumulate in damaged cartilage and deliver ghrelin mRNA, improving structural and pain-related outcomes in mice (32). This result demonstrates that delivery efficiency can depend on disease-associated matrix changes. For an efferocytosis-directed therapy, however, cartilage penetration cannot substitute for evidence of exposure in the intended macrophage population.

Drug residence time is another actionable variable. A hyaluronic acid–metformin conjugate retained detectable metformin in mouse knee tissues for up to seven days and improved experimental post-traumatic OA compared with less effective unconjugated formulations (33). These findings support sustained local delivery of a metabolic intervention, but neither prolonged retention nor cartilage protection establishes an efferocytosis-mediated mechanism. Carrier distribution, intracellular cargo availability, and clearance function should therefore be measured together.

### 5.3 Clinical translation and trial design

Clinical evidence supports investigating metabolic interventions in OA while maintaining a distinction between symptomatic benefit and mechanistic validation. In a randomized trial involving 107 participants with knee OA and overweight or obesity, oral metformin reduced pain at six months compared with placebo, with a between-group difference of −11.4 mm on a 100-mm visual analog scale. The trial did not establish restoration of synovial efferocytosis or structural disease modification (34). Thus, metabolic treatment provides a clinically relevant context for testing clearance-related mechanisms, rather than confirmation that these mechanisms mediate benefit.

Intra-articular gene therapy also offers translational lessons. A phase 1 study of PCRX-201, an inflammation-responsive IL-1 receptor antagonist gene therapy, reported limited extra-articular biodistribution and exploratory improvements in symptoms. Treatment-related knee effusions were common, and 45.8% of participants discontinued by week 104, limiting interpretation of the uncontrolled efficacy observations (35). This study informs local delivery and monitoring strategies but does not validate efferocytosis-directed treatment.

Future studies should combine patient stratification with measurements of target engagement and clearance function. Candidate assessments include macrophage uptake and degradation of apoptotic material, relevant receptor modifications, inflammatory mediator output, and imaging of synovitis, alongside pain, function, and structural outcomes. For multicomponent formulations, mechanistic perturbation experiments should establish whether the benefit depends on restored efferocytosis. Demonstrating this dependence will be essential to distinguish a treatment that corrects clearance failure from one that improves OA through parallel anti-inflammatory or chondroprotective effects.

## 6 Discussion and future perspectives

The evidence reviewed here supports a working model in which metabolic stress can compromise macrophage efferocytosis. This dysfunction may link systemic metabolic disturbances to persistent inflammation within the osteoarthritic joint. Establishing its therapeutic relevance requires resolving three interconnected uncertainties: which macrophage populations develop clearance defects, whether these defects causally contribute to disease progression, and which patients would benefit from their correction. **Box 1** outlines these questions and proposes experiments to address them.

Box 1 **Key unresolved questions and proposed experiments**

1. Which synovial macrophage populations develop efferocytosis defects?

Proposed experiments: Combine spatial profiling of human OA synovium with functional assays in matched, phenotypically defined macrophage subsets. In experimental OA, use lineage tracing to distinguish resident from recruited macrophages. Measure apoptotic-cell binding, internalization, lysosomal degradation, and sequential uptake separately. Where feasible, include apoptotic synovial cells alongside standardized targets. Quantify macrophage abundance and viability to distinguish loss of competent efferocytes from impaired function within surviving populations.

Key readout: A reproducible clearance defect localized to a defined macrophage population and efferocytic step, rather than inferred from receptor expression alone. Relevant evidence: (6, 36).

2. Does impaired efferocytosis causally contribute to OA progression?

Proposed experiments: Use macrophage-restricted perturbations and complementary rescue approaches in metabolic OA models to test candidate defects, including reduced GAS6 availability and CD11b lactylation. Determine whether clearance restoration protects joints despite persistent metabolic stress, and whether a separately validated perturbation that prevents clearance recovery attenuates this protection. Include controls for altered macrophage recruitment, survival, and efferocytosis-independent signaling. In matched macrophage assays, compare acute and sustained metabolic exposure to distinguish inhibitory CD11b lactylation from adaptive lactate-dependent continual efferocytosis.

Key readout: Recovery of complete clearance accompanied by reduced synovitis and cartilage damage, with diminished protection when clearance recovery is prevented. Measure apoptosis rates and macrophage inflammatory output to exclude reduced cell death or increased uptake without resolution as alternative explanations. Relevant evidence: (7, 8, 18, 20).

3. Which patients are most likely to benefit from efferocytosis restoration?

Proposed experiments: Establish longitudinal OA cohorts spanning disease stages and metabolic profiles. Integrate glycemic and lipid measures with synovial macrophage composition, local metabolic features, and direct clearance assays, accounting for sex, age, adiposity, and medication use. Test whether baseline clearance defects predict subsequent synovitis, pain, or structural progression. Where feasible, assess ex vivo responses to candidate interventions before evaluating a prespecified biomarker panel in an independent cohort and a stratified clinical trial.

Key readout: A reproducible patient subgroup with demonstrable clearance dysfunction. Predictive value for treatment selection would require evidence that baseline dysfunction modifies the response to an efferocytosis-directed intervention. Relevant evidence: (6, 39, 40).

Macrophage identity and anatomical context should guide this investigation. Recent fate-mapping studies in experimental OA distinguish expanding monocyte-derived TIM4-negative macrophages from resident populations that undergo spatial redistribution. Complementary human single-cell analyses identify a MERTK-low, CD48-high, CCR2-positive macrophage subset associated with oncostatin M signaling and fibroblast activation (36). These findings provide a framework for investigating clearance competence beyond conventional M1/M2 classifications. However, receptor expression and transcriptional signatures cannot establish efferocytic function. Spatial profiling should therefore be paired with measurements of apoptotic-cell internalization, cargo degradation, and repeated uptake in defined macrophage populations. Such analyses could distinguish loss of competent efferocytes from functional suppression within an otherwise preserved population.

Causal attribution also requires consideration of the surrounding stromal compartment. Human single-cell studies support an integrated infrapatellar fat pad–synovial tissue unit, with interactions among fibroblasts, adipocytes, and macrophages contributing to joint pathology (37). Moreover, work in diabetic OA identifies YAP1-regulated glycolysis in fibroblast-like synoviocytes as a driver of macrophage infiltration and polarization (38). Thus, improvements following metabolic intervention may reflect altered macrophage recruitment or stromal signaling without demonstrating restoration of macrophage efferocytosis. Cell-restricted perturbations and complementary rescue experiments are needed to establish whether clearance recovery mediates tissue protection. Particularly informative experiments would test whether restoring efferocytosis remains protective under persistent metabolic stress and whether preventing this restoration attenuates therapeutic benefit.

Patient stratification presents a further challenge. Multiomic profiling of the human infrapatellar fat pad reveals fibroblast transcriptional differences associated with sex and obesity, alongside obesity-associated differences in fibroblast-derived metabolites (39). These observations suggest that body mass index alone may inadequately represent the local metabolic environment relevant to macrophage function. Future cohorts should integrate systemic metabolic measures with synovial cellular composition and direct clearance assays. Longitudinal sampling, where feasible, would help determine whether impaired efferocytosis precedes worsening synovitis or emerges in response to established tissue damage. An efferocytosis-deficient OA endotype should therefore remain a testable hypothesis until its temporal stability and clinical relevance are demonstrated.

Clinical relevance must also extend beyond molecular normalization. In human knee OA, worse pain is associated with fewer immune-regulatory macrophages, exhaustion-associated features, and reduced phagocytic activity. The functional assay in that study measured bead uptake, providing evidence of macrophage dysfunction without directly establishing defective apoptotic-cell clearance (40). This distinction argues for pairing efferocytosis-specific measurements with pain, function, and structural outcomes in future studies. Tissue apoptotic-cell burden should be interpreted alongside cell-death rates, because fewer detectable corpses could reflect reduced apoptosis rather than improved clearance.

The translational value of this framework will depend on demonstrating that restoration of efferocytosis produces sustained inflammatory resolution in a reproducibly identifiable patient subgroup. Connecting macrophage identity, metabolic context, and functional recovery would provide a stronger basis for targeted intervention and clarify when defective clearance is a disease driver rather than a consequence of joint injury.
